# Whole-Genome Sequencing-Based Characterization of *Listeria monocytogenes* from Fish and Fish Production Environments in Poland

**DOI:** 10.3390/ijms21249419

**Published:** 2020-12-10

**Authors:** Kinga Wieczorek, Arkadiusz Bomba, Jacek Osek

**Affiliations:** 1 Department of Hygiene of Food of Animal Origin, National Veterinary Research Institute, Partyzantow 57, 24-100 Pulawy, Poland; josek@piwet.pulawy.pl; 2 Department of Omics Analyzes, National Veterinary Research Institute, Partyzantow 57, 24-100 Pulawy, Poland; Arkadiusz.Bomba@piwet.pulawy.pl

**Keywords:** *Listeria*, fish, fish production environment, WGS, cgMLST, cluster analysis

## Abstract

*Listeria monocytogenes*, an important foodborne pathogen, may be present in different kinds of food and in food processing environments where it can persist for a long time. In this study, 28 *L. monocytogenes* isolates from fish and fish manufactures were characterized by whole genome sequencing (WGS). Core genome multilocus sequence typing (cgMLST) analysis was applied to compare the present isolates with publicly available genomes of *L. monocytogenes* strains recovered worldwide from food and from humans with listeriosis. All but one (96.4%) of the examined isolates belonged to molecular serogroup IIa, and one isolate (3.6%) was classified to serogroup IVb. The isolates of group IIa were mainly of MLST sequence types ST121 (13 strains) and ST8 (four strains) whereas the isolate of serogroup IVb was classified to ST1. Strains of serogroup IIa were further subtyped into eight different sublineages with the most numerous being SL121 (13; 48.1% strains) which belonged to six cgMLST types. The majority of strains, irrespective of the genotypic subtype, had the same antimicrobial resistance profile. The cluster analysis identified several molecular clones typical for *L. monocytogenes* isolated from similar sources in other countries; however, novel molecular cgMLST types not present in the *Listeria* database were also identified.

## 1. Introduction

Listeriosis, caused by *Listeria monocytogenes*, has become a serious problem for human health. A statistically significant increasing trend in confirmed infection cases in the European Union (EU) has been observed since 2009 [1]. This situation is due to, among other things, the presence of *L. monocytogenes* in ready-to-eat (RTE) food, especially at the retail level, and the high pathogenic properties of the isolated bacteria [2]. Listeriosis is one of the most dangerous foodborne zoonoses because a high percentage of infected persons need to be hospitalized and due to the high mortality rate of the disease [1]. *L. monocytogenes* is able to contaminate a large variety of food, including RTE fish and fish products. These bacteria have the ability not only to survive but also to multiply under a wide range of different conditions and can persist for a long time in a food processing environment, thus, they are able to cross-contaminate final products at different production stages [3]. Furthermore, it has been shown that *L. monocytogenes* was responsible for multi-country outbreaks and that it survived in the food chain for several years [4,5].

Taking the above into account, it is essential to eliminate or at least to minimize the prevalence and dissemination of *L. monocytogenes* in food processing facilities. This may be achieved, e.g., by using effective disinfectants and microbiological monitoring of the food production environment and raw materials used for further food processing. Part of this strategy is reliable identification of the origin of the food, tracking of bacterial contamination, and comparison of the isolates from different sources. For these reasons, whole-genome sequencing (WGS), a rapid, sensitive, and accurate method, is now utilized in outbreak investigations, epidemiological studies, and surveillance programs worldwide [6,7,8]. The analysis of bacterial genome sequences provides detailed information on isolates’ relationships and reveals the place of the isolated strains in the international *L. monocytogenes* phylogenetic lineages and molecular types [9,10]. WGS also distinguishes between avirulent or hypovirulent and highly virulent strains on the basis of the lack or presence of pathogenic genes or genomic islands [7,11]. One of such most important molecular markers are genes grouped into *Listeria* pathogenicity islands (LIPIs), including LIPI-1, which contains the key genes responsible for the virulence of *L. monocytogenes*, i.e., the proliferation and intra- and intercellular bacterial movement [12]. Other LIPIs are of the internalin family of genes, which support *L. monocytogenes* in adhering to and invading host cells [13]. LIPI-3 is mainly identified in the bacteria of lineage I and encodes listeriolysin S (LLS), expressed by *L. monocytogenes* in the gastrointestinal tract during infections [14]. Recently, the LIPI-4 gene cluster associated with the hypervirulent strain of clonal complex CC4, responsible for the bacterial tropism to the central nervous system, has been described among human *L. monocytogenes* isolates [7].

WGS-based data are very useful for the classification of the isolates as persistent, i.e., which are present in a food processing environment for a long time, even for years. The reason for this phenomenon is not fully understood, but some factors having an influence on the persistence of specific *L. monocytogenes* molecular variants have been described [15]. Among them are genes responsible for resistance to quaternary ammonium compounds (QAC), including the benzalkonium chloride (BC) disinfectant applied in the food industry [16]. Additionally, the persistence of the bacteria is also associated with the presence of the *comK* gene, which is involved in biofilm development [17,18]. Other markers, which support *L. monocytogenes* survival and persistence in suboptimal environmental conditions such as alkaline and oxidative stresses, are the five-gene stress survival islet 1 (SSI-1) and the protease gene regulated by the transcription factor under stress conditions SSI-2 [19,20].

In the present study, 28 *L. monocytogenes* isolates from fish and fish-production environments in Poland were characterized using WGS to establish molecular clones and then compared with the strains from other countries. Additionally, the virulence and antimicrobial resistance genotypes of the isolates were identified, with particular emphasis on the genes encoding resistance to disinfectants.

## 2. Results

### 2.1. Population Structure of L. monocytogenes Isolates Tested

A total of 28 *L. monocytogenes* isolates were obtained from 490 samples tested. Among them, six isolates were from raw salmon, 14 strains from cold-smoked salmon, and eight isolates from a fish production environment. Molecular typing showed that all but one (27; 96.4%) of the examined strains belonged to molecular serogroup IIa and only one isolate (3.6%) was classified to serogroup IVb. The latter strain, recovered from raw salmon, was further subtyped to lineage I (LI), sublineage 1 (SL1), MLST sequence type 1 (ST1), and the novel cgMLST type CT7225. Twenty-seven *L. monocytogenes* isolates of serogroup IIa clustered into one group with 13 strains were classified to ST121, whereas the remaining IIa isolates belonged to ST8 (four strains), ST155 and ST173 (three strains of each), ST31 (two strains), and ST7 and ST504 (one strain of each) (Table 1 and Table S3).

**Figure 1:**
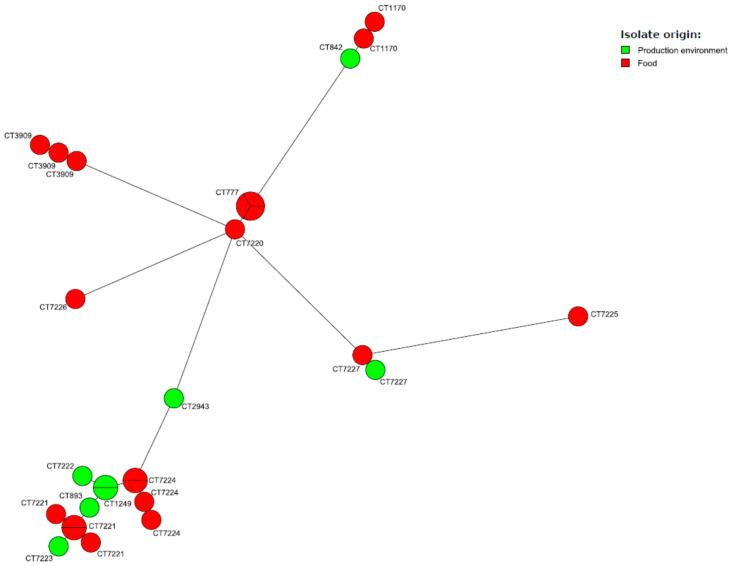
MST of cgMLST allelic profiles of 28 *L. monocytogenes* studied strains. cgMLST types (CTs) are represented by colored circles where size is proportional to the number of isolates.

Further molecular subtyping with the cgMLST method revealed that the 27 *L. monocytogenes* IIa strains were classified into seven different sublineages, with the most numerous being SL121 (13; 48.1% strains) and SL8 (four; 14.8% strains). Strains of SL121 belonged to six cgMLST types, mainly CT7221 and CT7224 (four strains of each), which were submitted as new cgMLST types to the *Listeria* PasteurMLST database. CT7221 strains were isolated from raw salmon in one fish manufacturer, whereas strains of CT7224 were recovered from cold-smoked salmon, also from one fish establishment but different to that of the CT7221 strains. *L. monocytogenes* classified into the second most numerous sublineage SL8 covered the strains of two different cgMLST types, i.e., CT777 (three isolates) and CT7720 (one strain); the latter one was identified as a new type. Both CTs originated from smoked salmon from two different fish manufacturers. Detailed information on the molecular subtypes of all *L. monocytogenes* strains tested is shown in Table S3, while their genetic relatedness based on cgMLST is presented in Figure 1.

### 2.2. Antimicrobial Resistance and Virulence Gene Profiles

Screening of *L. monocytogenes* WGS data for 17 antimicrobial resistance genes revealed that almost all (26; 92.9%) of the strains had the same resistance profile with the presence of the *fosX* (fosfomycin), *lmo0919* (antibiotic ABC transporter ATP-binding protein), *mprF* (phosphatidylglycerol lysyltransferase), *norB* (multidrug efflux pump), and *sul* (dihydropteroate synthases) genes. Acquired antibiotic resistance traits were not detected, with the exception of the *aacA4* gene responsible for resistance to aminoglycosides, which was present in two isolates belonging to cgMLST type CT7227.

The LIPI-1 virulence genes were identified in all 28 isolates, whereas LIPI-4 was not found in any of the *L. monocytogenes* studied. Additionally, the presence of LIPI2_inlII (LIV_RS06070) was observed in all strains of serogroup IIa. The LIPI-3 genes were only associated with one strain of serogroup IVb belonging to lineage I. The internalin *inlABCEHJK* markers were detected in all isolates, but the *inlF* and *inlG* genes were found in 14 and 11 *L. monocytogenes* tested, respectively (Table 2). The premature stop codon (PMSC) was harbored by strains belonging to sublineages SL121 (13 isolates, PMSC mutation type 6, [21]) and SL31 (two strains, PMSC mutation type 5, [22]) (Table S3).

Furthermore, all *L. monocytogenes* were positive for the presence of intracellular survival, regulation of transcription and translation, surface protein anchoring, peptidoglycan modification, and immune modulation as well as bile salts resistance genes, respectively. The occurrence of some markers was correlated with *L. monocytogenes* serogroups. The *gltA* and *gltB* genes involved in teichoic acid biosynthesis were found in the only strain belonging to serogroup IVb, whereas the *tagB* gene, also engaged in the same process, was identified in all 27 strains classified to serogroup IIa. Additionally, the *ami* (adherence) and *aut* (invasion) gene markers were observed among all strains of IIa serogroup, whereas the *aut_IVb* gene (autolysin) was present in the only *L. monocytogenes* isolate classified to serogroup IVb. Detailed information on the virulence and antimicrobial resistance gene profiles are shown in Table 2 and Table S3.

### 2.3. Benzalkonium Chloride and Other Tolerance Genes

The *bcrABC* gene cassette, encoding benzalkonium chloride resistance protein, *emrE* marker, responsible for putative small multidrug-resistant (SMR) efflux pump, and the *qacA* gene, encoding for quaternary ammonium compound efflux major facilitator superfamily (MFS), were not identified in the sequences of any *L. monocytogenes* tested. Twelve of 28 (42.9%) strains harbored the *Tn6188*_qac (*ermC*) sequence responsible for tolerance to benzalkonium chloride. The *cadA* gene, encoding for cadmium resistance protein, was present in only one isolate of serogroup IIa classified to cgMLST type CT1170. All five genes of the stress survival islet 1 (SSI-1) were observed in the 10 *L. monocytogenes* IIa strains, belonging to six different cgMLST types, whereas the SSI-2 genes were found in 14 isolates of serogroup IIa, mainly classified to the sequence type ST121 (13 strains) and belonging to seven various CTs (Table 2 and Table S3). Additionally, all 28 strains showed the presence of other stress response genes, e.g., encoding for acid (*gadT2* and from *lmo0036* to *lmo0043*), temperature (*cspB*), and water (*gbuA*, *gbuC*, *opuCA*, *opuCC*, *opuCD*, *betL*) tolerances, respectively. Furthermore, all *L. monocytogenes* had the *comK* gene, connected with virulence and biofilm formation as well as the *mdrL* marker and *sigB* operon, responsible for multidrug transporter and various stress cell responses, respectively.

### 2.4. Identification of Prophage Sequences

DNA sequence analysis of 28 *L. monocytogenes* isolates tested identified from two to seven prophage regions, including intact prophage sequences found in 25 (89.3%) strains, with the most common sequence being PHAGE_Lister_LP_101 (20 isolates). The prophage sequences integrated with the *comK* gene were present in the genome of 22 strains classified to five different sequence types, but the sequence was intact in only two strains (belonging to ST7 and ST121). These two *L. monocytogenes* isolates were isolated from frozen salmon from the same fish manufacturer but from different product lots (Table S3). None of the prophage sequences was found within the *comK* gene harbored by six strains classified to ST173 (three isolates), ST31 (two strains), and ST1 (one strain). Detailed information on the predicted *L. monocytogenes* prophage sequences are presented in Table S4.

### 2.5. Comparison of L. monocytogenes Sequences from Different Sources

**Figure 2:**
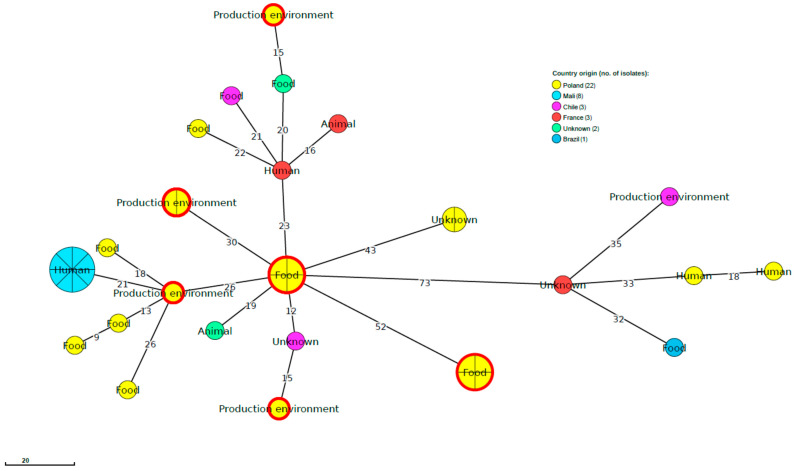
MST based on the cgMLST profiles of 13 *L. monocytogenes* strains studied belonging to SL121 together with 26 SL121 strain profiles publicly available at BIGSdb-Lm (listed in Table S2). cgMLST are represented by colored circles where size is proportional to the number of isolates. Numbers on the branches show alleles differences between neighboring nodes. Circles with red rim represent *L. monocytogenes* strains from the present study.

**Figure 3:**
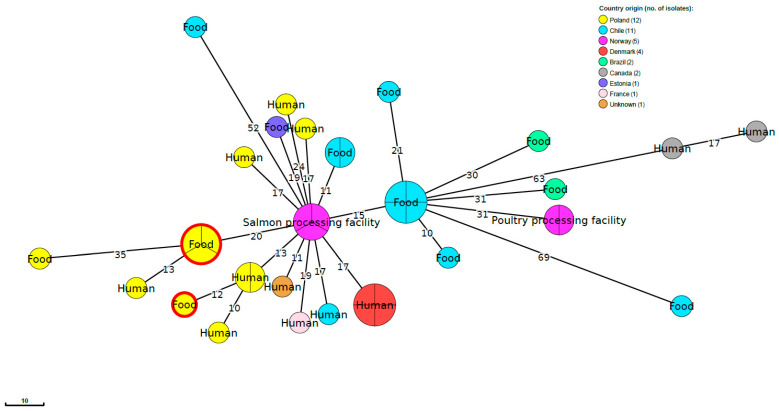
MST based on the cgMLST profiles of 4 *L. monocytogenes* of SL8 identified in the present study together with 35 SL8 strain profiles publicly available (listed in Table S2). cgMLST are represented by colored circles where size is proportional to the number of isolates. Numbers on the branches show alleles differences between neighboring nodes. Circles with red rim represent *L. monocytogenes* strains from the present study.

Comparative molecular analysis of the current 13 *L. monocytogenes* strains classified to SL121 and the genome of 26 isolates of the same cgMLST sublineage publicly available in the BIGSdb-Lm database did not reveal any close relationships between our strains and isolates from other countries, including previously identified strains in Poland (Figure 2). None of the compared isolates were classified to the same CTs as the *L. monocytogenes* identified in the present study. The same was observed when four strains of SL8 were compared with the sequences of 16 *L. monocytogenes* of human origin, including seven strains from Poland. This phylogenetic analysis indicated that the strains identified in the present study were not molecularly related to previously sequenced *L. monocytogenes*. Furthermore, none of the strains from humans were classified to the same cgMLST type as the isolates from fish and fish production environments analyzed in the current investigation (Figure 3).

## 3. Discussion

In this study, WGS was used for the characterization of *L. monocytogenes* isolated from fish and fish processing environments, including the identification of genes putatively responsible for the persistence features of the isolates. The two most common molecular types, i.e., sequence types ST121 and ST8, were found among 17 out of 28 (60.7%) *L. monocytogenes* isolates tested. These strains were classified into clonal complexes CC121 and CC8, different from the most common CCs found among the clinical isolates responsible for listeriosis cases in Europe [14]. However, strains of CC8 were connected with human infections in China and Canada; therefore, the virulence potential of such strains must not be underestimated [23,24]. A relatively frequent (46.4%) *L. monocytogenes* CC121 identified in the present study was previously only sporadically detected among clinical isolates in Poland (two out of 344; 0.6% strains), whereas isolates classified to CC8 (14.3% of the current strains) were recovered from humans with listeriosis more often (n = 21; 6.1%) [25]. However, in the mentioned study, 190 (55.2%) of the *L. monocytogenes* strains isolated from humans with listeriosis belonged to serogroup IVb, which was currently identified in only one isolate from fish, which may suggest that such food was not a source of those infections [25]. Furthermore, the phylogenetic comparison of the studied *L. monocytogenes* SL121 with other strains classified to this sublineage identified globally did not reveal any close genetic relationship of the isolates. Such strains have been previously designated as the hypovirulent food-associated clone and were identified among the six most prevalent STs of food and food processing plant origins, being also responsible for sporadic listeriosis cases in Europe [14,26,27,28]. The hypovirulent properties of ST121 strains are also supported by the fact that all isolates tested in the present investigation possessed the PMSC codon in the *inlA* gene that is associated with reduced bacterial pathogenicity [6]. On the other hand, the current *L. monocytogenes* strains belonging to ST121 harbored several other pathogenic determinants that are involved in the bacteria’s virulence and listeriosis development (Table S3). Rychli et al. (2017) also revealed that ST121 strains with the truncated *inlA* gene were isolated from clinical cases, so they might have other mechanisms to cause listeriosis in humans [29]. Furthermore, cgMLST analysis of *L. monocytogenes* ST121 strains allowed to identified six different cgMLST types, of which four were novel. This may suggest that such strains were not previously isolated or such sequences were not submitted to the PasteurMLST BIGSdb-*Lm* database.

The presence of LIPI-1 and the internalin family *inlABCEFGHJK* genes was observed in all or in almost all tested *L. monocytogenes*, respectively. Among the isolates classified to ST121 and ST31, the truncated internalin A gene was identified, which may indicate their reduced virulence properties, whereas other *L. monocytogenes* analyzed, including those classified to ST8, possessed the full-length *inlA* gene. Previous studies also suggested that isolates with the PMSC mutation were over-represented in food and food production environments, which was not confirmed in the current study [10,30]. However, a recent study conducted by Chen et al. (2020) indicated a significant shift towards a higher proportion of *L. monocytogenes* of food origin with the intact *inlA* gene, which indicates their higher virulence potential [31].

The LIPI-3 pathogenic island, characteristic for *L. monocytogenes* hypervirulent clones, was identified in only one strain belonging to the IVb serogroup. It was previously shown that this gene cluster was strongly associated with IVb strains, which was also confirmed in the current study, although only one such isolate was identified [32]. Furthermore, it has also been described that hypervirulent *L. monocytogenes* strains were connected with the presence of LIPI-4, which was not found in any of the examined isolates [14,31]. This pathogenic island was often associated with strains of clonal complex CC4, which were not identified in the present investigation.

As shown here, none of the 28 *L. monocytogenes* isolates possessed the *bcrABC* gene cassette, encoding for resistance to benzalkonium chloride (BC), which may suggest that these strains were sensitive to BC. This may be due to, e.g., a lack of selection pressure for such strains from disinfectants containing BC or isolation of the bacteria from hard-to-clean equipment or surfaces where they had limited contact with this compound [33]. Some investigators have also suggested that BC-resistant strains were characterized by a lower ability to produce biofilm compared to susceptible isolates [34]. However, it has been previously shown that it is not only the *bcrABC* genes that are responsible for resistance to quaternary ammonium compounds (QAC) [35]. The *Tn6188* transposon involved in bacterial resistance to BC, was identified in 42.9% of *L. monocytogenes* tested, including nine out of 13 isolates classified to ST121, and such strains were also found by other authors [7,28,29]. A statistically significant correlation between the presence of the *emrC* gene and *L. monocytogenes* SL121 subtype was observed by Moura et al. (2016). This gene possesses a partial homology to the quaternary ammonium efflux gene located on the *Tn6188* transposon, and it is responsible for resistance to BC [36,37]. It has been suggested that the *emrC* marker is one of the bacterial factors which has an influence on the persistence of the *L. monocytogenes* SL121 clonal type in food processing plants. However, Cherifi et al. (2018) recommended that not only one genetic determinant should be taken into consideration when strain persistence is investigated. It has been shown that *L. monocytogenes* isolates susceptible to BC had better abilities to form a biofilm, which in total offers more possibilities to survive in food production environments.

It has been previously described that the persistence of some *L. monocytogenes* strains in food establishments was also related to the presence of other molecular traits, e.g., the stress survival islets SSI-1 and SSI-2 [19,20]. In the present study, the SSI-2 marker was identified in all *L. monocytogenes* SL121 isolates, which suggests their better adaptation to the alkaline and oxidative stress conditions often present in fish processing environments. Furthermore, the SSI-1 genes play a role in bacterial survival and even growth under gastric stress conditions, and they have often been identified in clinical isolates responsible for listeriosis in humans [19]. In the present study, all SSI-1 islet genes were identified in 10 strains, including *L. monocytogenes* classified to clonal complexes CC8 and CC155, which may suggest their higher pathogenic potential for human infections.

*L. monocytogenes* sequence type ST8 has also been previously described as a persistent variant [38,39]. Such strains were also isolated during a cross-border outbreak of listeriosis caused by cold-smoked salmon in Denmark and France and were of cgMLST type CT771 [8]. During the present study, four *L. monocytogenes* ST8 isolates belonging to CT777 (three strains) and CT7220 (one strain) were identified, but none were closely related to the strains previously isolated from humans, whose sequences are available in the BIGSdb-Lm database. Furthermore, the *L. monocytogenes* ST8 currently recovered from fish were genetically different than those described in the EFSA and ECDC ROA document on the multi-country listeriosis outbreak during 2014–2019 linked to the consumption of cold-smoked fish products [5]. It has been previously shown that *L. monocytogenes* ST8 strains had a strong ability to persist in food processing plants, including smoked fish production environments [8,38,39]. Furthermore, such isolates were also transferred between different food enterprises through raw food materials [39]. This may result in the persistence of certain *L. monocytogenes* clones in environmental niches for a long time and their transmission between food facilities [39,40]. Finally, this may interfere with the interpretation of data from WGS analyses performed during outbreak investigations [41,42].

The presence of antimicrobial-resistant *L. monocytogenes* in food and food production environments may lead to an urgent threat to public health. In the present study, the WGS analysis has shown that the isolates tested had very similar antibiotic resistance gene profiles. All of them were positive for the efflux pump-related *mdrL* and *norB* markers, responsible for resistance to macrolides and fluoroquinolones, respectively [35,43]. Interestingly, the *norB* gene, which confers resistance to certain quinolones, such as moxifloxacin, has been identified in *Staphylococcus aureus* strains, but it is rather uncommon in *Listeria* isolates [44]. However, the role of this gene marker in the pathogenesis of human listeriosis and resistance to quinolones remains unclear. Furthermore, none of the 28 *L. monocytogenes* isolates tested in the present study were positive for the tetracycline resistance *tetM* marker, a ribosomal protection gene related to the presence of the conjugative transposon Tn916 [45,46]. This gene was previously commonly identified in *L. monocytogenes* of food origin isolates resistant to tetracyclines [47,48,49].

Several sequences encoding the presence of prophages were detected among 28 *L. monocytogenes* isolates tested, including intact, questionable, or incomplete molecular markers (Table S4). These prophages were originated from different *Listeria* phages such as LP101, A006, and LmoS188, and were commonly found in *Listeria* strains by other authors [32,50]. It has been previously described that the presence of prophage genes in *L. monocytogenes* had an influence on bacterial virulence and their persistence in food processing environments as well as intracellular survival in the human host [17,18]. Especially, phages inserted into the *comK* gene, which encodes functional protein ComK required for *L. monocytogenes*’ intracellular growth, provide better adaptation to food production environments through, e.g., biofilm formation [18,28]. In the present study, such a prophage sequence inserted into the *comK* gene was identified in 22 strains; however, in only two cases, the intact prophage genome was found. Furthermore, no correlation between *L. monocytogenes* sequence types and the presence of prophage sequences was observed. The lack of such a relationship was also identified by Knudsen et al. (2017), who suggest that the insertion of prophage sequences into the *L. monocytogenes* genome may have an influence on the molecular variability of the isolates [51].

## 4. Materials and Methods

### 4.1. L. monocytogenes Isolation

All samples tested for *L. monocytogenes* were collected during the first quarter of 2019 from 16 fish manufacturers located in Poland, which were selected based on fish production volume (more than five tons per month). The isolates investigated in the present study were the results of the target-specific sampling ordered by Chief Veterinary Officer on 27 February 2019 in cold-smoked salmon establishments in Poland. The samples tested in the present study included raw fish material, i.e., fresh and frozen Atlantic salmon (*Salmo salar*) (n = 130), swabs from fish production environments (n = 200) as well as samples from ready-to-eat (RTE) fish products, i.e., cold-smoked Atlantic salmon (n = 90), marinated (gravlax) Atlantic salmon (n = 45), and cold-smoked rainbow trout (*Oncorhynchus mykiss*) (n = 25). The number of samples taken from each plant was different but representative of the production quantity of each manufacturer. The samples were tested towards *L. monocytogenes* isolation in official veterinary laboratories using the ISO 11290-1:2017 method [52]. Then, the bacterial isolates were sent to the National Reference Laboratory for *L. monocytogenes* located at the Department of Hygiene of Food of Animal Origin in the National Veterinary Research Institute in Pulawy. After confirmation, the *L. monocytogenes* isolates were stored at −70 °C for further analysis.

### 4.2. DNA Isolation, Library Preparation, and Sequencing

*L. monocytogenes* was cultured on tryptone soya-yeast extract agar (TSYEA; Oxoid, Basingstoke, UK) at 37 ± 1 °C for 18–24 h and then a loopful of bacteria was transferred into 100 µL of 10 mM TRIS-HCl buffer, pH 8.5 (Sigma-Aldrich, St. Louis, MO, USA). Next, 15 µL of lysozyme (10 mg/mL; Sigma-Aldrich) was added and the samples were incubated at 37 °C for 30 min. DNA was extracted using a Maxwell^®^RSC 48 automated nucleic acid purification platform (Promega, Madison, WI, USA) and a Maxwell^®^RSC Cultured Cells DNA kit (Promega). DNA quality and concentration were measured by a NanoDrop and a Qubit 3 (Thermo Fisher Scientific, Waltham, MA, USA). A DNA library was prepared with the Nextera XT DNA Library Preparation Kit (Illumina, San Diego, CA, USA) according to the manufacturer’s instruction. DNA was sequenced using Illumina MiSeq sequencing platforms (Illumina).

The libraries were then sequenced on the Illumina MiSeq platform (2 × 300 reads) at approximately 50× average coverage. De novo genome assembly was performed using SPAdes 3.9.0 [53] on trimmed reads with Trimmomatic 0.36 [54]. The genomes were assembled in several contigs ranging from 13 to 27 (mean 19.8), with a mean coting length of 158,234.7 and a mean N50 value of 665,789.2. The detailed sequence parameters are listed in Table S1.

All *L. monocytogenes* DNA sequences were deposited in the *Listeria* PasteurMLST database (https://bigsdb.pasteur.fr/listeria) under the accession numbers listed in Table S1.

### 4.3. Molecular Typing of L. monocytogenes Using WGS

PCR-based serogroups (five loci) [9], MLST (7 loci) [55], and cgMLST profiles (1748 loci) [6] were extracted from the assemblies using the tool available on the BIGSdb-Lm platform (https://bigsdb.pasteur.fr/listeria) and the chewBBACA 2.1.0 software on the same scheme [56].

MLST profiles with the same alleles for seven loci were classified into sequence types (ST) and grouped into clonal complexes (CCs) if at least five out of seven loci were the same as previously described [55]. cgMLST profiles were grouped into cgMLST types (CTs) and sublineages (SLs), using the cut-offs of seven and 150 allelic mismatches, respectively, as previously described [7].

### 4.4. WGS Identification of Virulence, Antimicrobial Resistance and Stress-Related Genes

*L. monocytogenes* 92 virulence genes, including the internalin, LIPI-1, LIPI-2, LIPI-3, and LIPI-4 genes, were detected using the virulence schemes available in the BIGSdb-Lm (https://bigsdb.pasteur.fr/listeria) and Abricate v.1.0.1 software (GitHub, San Francisco, CA, USA) [7]. Antimicrobial resistance genes were identified in silico from the WGS data using the 17 loci scheme including such markers as *aacA4*, *ermB*, *fosX*, and *tetM* [7]. Furthermore, the presence of metal and detergent resistance genes (eight loci scheme) [7], stress island markers (seven loci) [19,20], and genes responsible for stress response, e.g., osmotolerance proteins, were also tested using the BIGSdb-Lm platform and Abricate v.1.0.1 software (GitHub).

### 4.5. Determination of L. monocytogenes Presumptive Persistence Genes

The sequence analysis of the *sigB* operon genes (eight loci), *mdrL* and *comK* genes was performed using the BIGSdb-Lm platform. The *Tn6188* transposon (*ermC*) and *cadA* gene are part of the metal- and detergent-resistance schemes mentioned in point 2.4. The presence of phage genes was investigated using PHASTER [57,58]. The criteria for scoring prophage regions (as intact, questionable, or incomplete) have been described in PHASTER [57]. If the region’s total score was less than 70, it was marked as incomplete, between 70 to 90 as questionable, and when greater than 90 defined as intact.

### 4.6. Comparison of L. monocytogenes with Isolates from Humans and Food

The WGS sequences of the isolates tested in the present study were compared with the sequences of *L. monocytogenes* classified to SL121 and SL8 sublineages recovered from other sources to assess their phylogenetic relationship. For this comparison, all SL121 isolates, irrespective of the source, and SL8 strains from human listeriosis cases were selected from the BIGSdb-Lm database. Additionally, eight *L. monocytogenes* strains belonging to sublineage SL8 were selected from the available literature [8,39]. To create a phylogenetic tree, cgMLST profiles of the selected strains were compared using the GrapeTree software and MSTreeV2 algorithm [59]. Detailed information on the *L. monocytogenes* strains tested is listed in Table S2.

## 5. Conclusions

The WGS analysis performed in the present study revealed the genetic diversity of *L. monocytogenes* from fish and fish processing areas in Poland. Several molecular clones identified were typical for the strains isolated from similar sources in other parts of Europe. On the other hand, the cgMLST analysis detected novel molecular types that were not present in the *Listeria* database. All isolates classified as ST121 and ST31 revealed a mutation in the *inlA* gene, which categorized them as hypovirulent strains. In contrast, *L. monocytogenes* of the IVb and IIa serogroups, belonging to clonal complexes CC1 and CC8, respectively, had the characteristics of hypervirulent isolates. According to our knowledge, this is the first study on WGS analysis of *L. monocytogenes* from fish and fish production environment in our country. The obtained data may be useful for epidemiological investigations connected to this foodborne pathogen. There is a need to provide more genetic information on *L. monocytogenes* from various countries for bacterial source identification and tracking of these microorganisms along the food chain. The present studies underlined the necessity of molecular typing of the bacteria from food and food production areas as well as the implementation of effective hygienic procedures to prevent contamination of food with *L. monocytogenes*.

## Figures and Tables

**Table 1:** Molecular characteristic of *L. monocytogenes* strains tested.

Strain ID	Source	Molecular Characteristics				
		PCR-Serogroup	ST	CC	SL	cgMLST
46894	P	IIa	155	CC155	SL155	CT842
46895	P	IIa	504	CC475	SL475	CT2943
46896	P	IIa	121	CC121	SL121	CT1249
46897	P	IIa	121	CC121	SL121	CT1249
46898	F	IVb	1	CC1	SL1	CT7225 *
46899	F	IIa	8	CC8	SL8	CT7220 *
46900	F	IIa	8	CC8	SL8	CT777
46901	F	IIa	8	CC8	SL8	CT777
46902	F	IIa	8	CC8	SL8	CT777
46903	P	IIa	121	CC121	SL121	CT893
46904	F	IIa	121	CC121	SL121	CT7221 *
46905	F	IIa	121	CC121	SL121	CT7221 *
46906	F	IIa	7	CC7	SL7	CT7226 *
46907	F	IIa	121	CC121	SL121	CT7221 *
46908	P	IIa	121	CC121	SL121	CT7223 *
46909	P	IIa	31	CC31	SL31	CT7227 *
46910	P	IIa	121	CC121	SL121	CT7222 *
46911	F	Ia	121	CC121	SL121	CT7221 *
46912	F	IIa	173	CC19	SL378	CT3909
46913	F	IIa	155	CC155	SL155	CT1170
46914	F	IIa	155	CC155	SL155	CT1170
46915	F	IIa	173	CC19	SL378	CT3909
46916	F	IIa	173	CC19	SL378	CT3909
46917	F	IIa	121	CC121	SL121	CT7224 *
46918	F	IIa	121	CC121	SL121	CT7224 *
46919	F	IIa	121	CC121	SL121	CT7224 *
46920	F	IIa	121	CC121	SL121	CT7224 *
46921	F	IIa	31	CC31	SL31	CT7227 *

P, fish production environment; F, RTE fish; ST, sequence type; CC, clonal complex; SL; sublineage; cgMLST, core genome MLST type; * new cgMLST type.

**Table 2:** Presence of selected genes among *L. monocytogenes* strains tested.

Strain ID	Presence of Genes								
	*inlA*	*inlF*	*inlG*	*aacA4*	*cadA*	*Tn6188*	LIPI-3	SSI-1	SSI-2
46894	1	1	1					1	
46895	1								1
46896	1 *					1			1
46897	1 *					1			1
46898	1	1					1		
46899	1	1	1			1		1	
46900	1	1	1					1	
46901	1	1	1					1	
46902	1	1	1					1	
46903	1 *					1			1
46904	1 *					1			1
46905	1 *					1			1
46906	1	1	1					1	
46907	1 *					1			1
46908	1 *					1			1
46909	1 **	1		1		1		1	
46910	1 *					1			1
46911	1 *					1			1
46912	1	1	1						
46913	1	1	1		1			1	
46914	1	1	1					1	
46915	1	1	1						
46916	1	1	1						
46917	1 *								1
46918	1 *								1
46919	1 *								1
46920	1 *								1
46921	1 **	1		1		1		1	

* premature stop codon type 5, ** premature stop codon type 6.

## Supplementary Materials

Supplementary materials can be found at https://www.mdpi.com/1422-0067/21/24/9419/s1. Table S1: L. monocytogenes sequence parameters, Table S2: Characteristics of L. monocytogenes strains selected for comparison with the current isolates, Table S3: Characteristics of L. monocytogenes tested, Table S4: Listeria *monocytogenes* prophages characteristics.

## Abbreviations

BC	benzalkonium chloride
BIGSdb-Lm	bacterial isolate genome sequence database *L. monocytogenes*
CC	clonal complex
cgMLST	core genome multilocus sequence typing
CT	core genome MLST complex type
LIPIs	*Listeria* pathogenicity islands
MLST	multilocus sequence typing
PMSC	premature stop codon
QAC	quarternary ammonium
RTE	ready to eat
SL	sublineage
ST	sequence type
WGS	whole-genome sequencing
